# Quadruple-Chamber Clot in Transit Traversing a Patent Foramen Ovale: Successful Percutaneous Mechanical Thrombectomy

**DOI:** 10.1016/j.jaccas.2026.109871

**Published:** 2026-08-26

**Authors:** Mitchell Mehringer, Blaine Massey, Mahmoud Ali, Bradley Clark

**Affiliations:** a Department of Internal Medicine, Ascension St. Vincent, Indianapolis, Indiana, USA; b Department of Cardiology, Ascension St. Vincent, Indianapolis, Indiana, USA; c Department of Vascular and Interventional Radiology, Ascension St. Vincent, Indianapolis, Indiana, USA

**Keywords:** atrial septal defect, echocardiography, pulmonary circulation, shortness of breath, three-dimensional imaging, thrombus, treatment

## Abstract

**Background:**

Clot in transit (CIT) is a rare, life-threatening manifestation of venous thromboembolism. When a thrombus traverses a patent foramen ovale, the risk of systemic embolization increases, and optimal management remains undefined.

**Case Summary:**

A 73-year-old woman presented with dyspnea and saddle pulmonary embolism with right heart strain. Echocardiography revealed a serpentine thrombus extending from the right atrium across the tricuspid valve, traversing a patent foramen ovale into the left atrium, and prolapsing through the mitral valve. After multidisciplinary discussion, she underwent mechanical thrombectomy with removal of a 14 cm intracardiac thrombus and pulmonary emboli. She recovered without clinically apparent systemic embolization.

**Discussion:**

Quadruple-chamber CIT with transseptal extension is exceedingly rare and carries high embolic risk. This case highlights mechanical thrombectomy as an alternative to surgery in selected patients.

**Take-Home Message:**

Mechanical thrombectomy may represent an effective and less invasive option for complex biatrial and biventricular CIT with pulmonary embolism.

## Visual Summary

**Figure:**
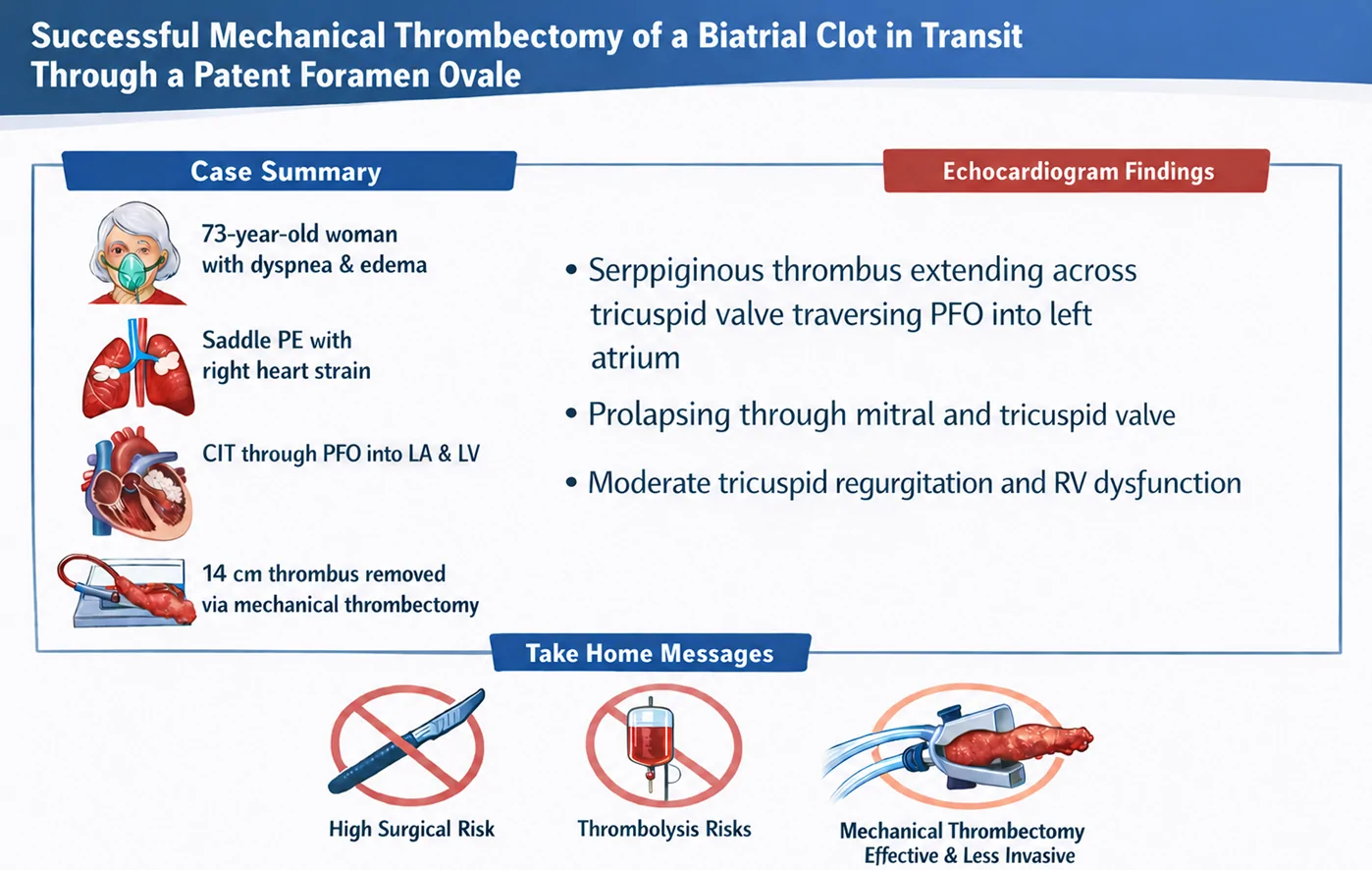

## History of Presentation

A 73-year-old woman presented initially to the outpatient office with worsening exertional dyspnea and lower extremity edema. She presented in no acute distress and denied shortness of breath at rest. Vital signs were notable for heart rate 106 beats/min, respirations 22 breaths/min, temperature 36.8 °C, blood pressure 160/85 mm Hg, and oxygen saturation 96% on room air. Physical examination revealed a regular, tachycardic rate without murmurs, clear lungs with normal work of breathing, and bilateral nonpitting lower extremity edema. She was alert and oriented to person, place, and time. Cranial nerves II to XII were intact, and her motor strength was 5/5 in all extremities.

## Past Medical History

Her history was significant for asthma and a remote history of breast cancer (29 years earlier). She denied a personal history of deep venous thrombosis (DVT) or pulmonary embolism (PE). However, her family history was notable for DVT in her mother and a fatal PE in her brother.

## Differential Diagnosis

Her differential diagnosis on admission was worrisome for heart failure exacerbation, PE, or asthma exacerbation.

## Investigations

Before her cardiology evaluation, a pro-B-type natriuretic peptide was obtained because of her dyspnea and edema and returned elevated at 5,750 pg/mL. This prompted her outpatient transthoracic echocardiogram, which demonstrated a preserved ejection fraction of 55%-60%, diastolic interventricular septal flattening consistent with right ventricular (RV) pressure overload, and a 6 × 1.2-cm mass in the right atrium (RA) (Figure 1). The mass extended from the RA to the left atrium (LA) through a patent foramen ovale, and prolapsed across the tricuspid and mitral valves with imaging indicating moderate tricuspid regurgitation and moderately reduced RV function (Video 1).

**Figure 1:**
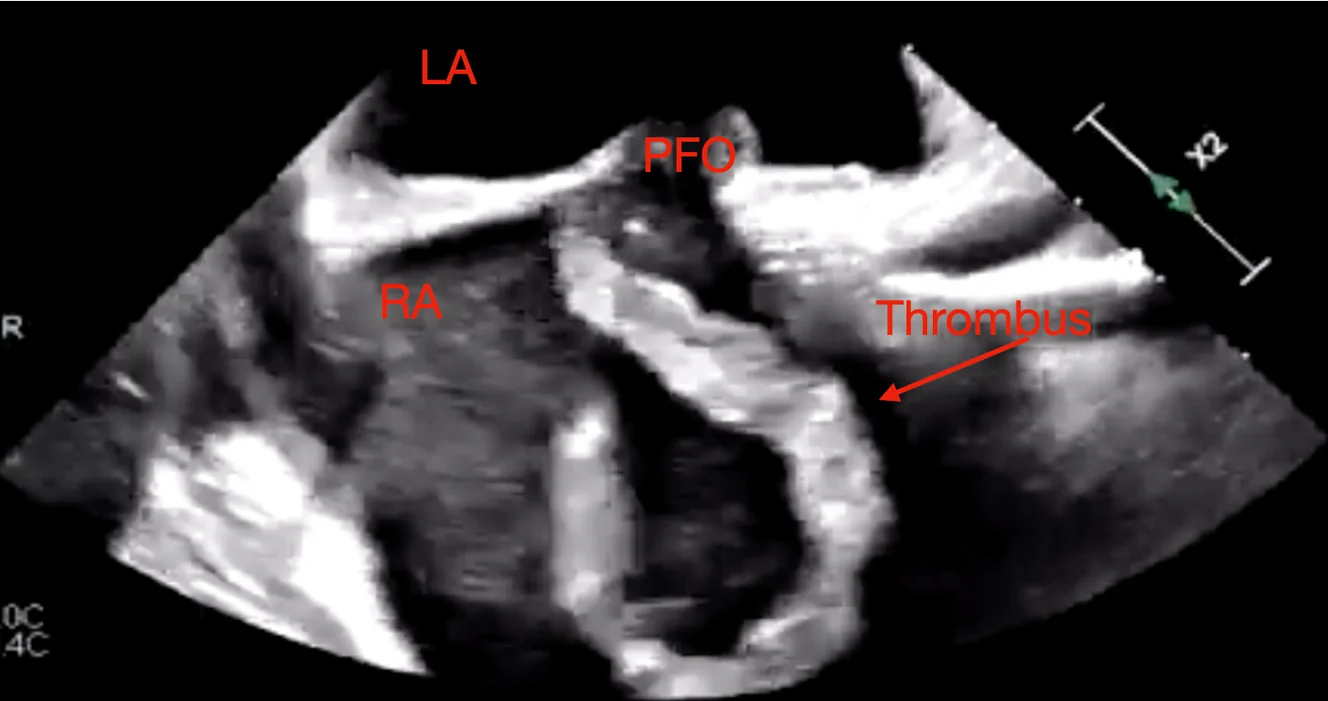
Transesophageal Echocardiogram Bicaval View of a RA Clot Extending Through a PFO LA = left atrium; PFO = patent foramen ovale; RA = right atrium.

Transesophageal echocardiogram (TEE) again further characterized a biatrial thrombus extending through a patent foramen ovale (PFO) (Video 2, Figure 2). A serpiginous clot was visualized in the RA with intermittent prolapse through the tricuspid valve, and extension across a PFO into the LA (Video 3, Figure 3). A bubble study demonstrated right-to-left shunting consistent with a PFO.

**Figure 2:**
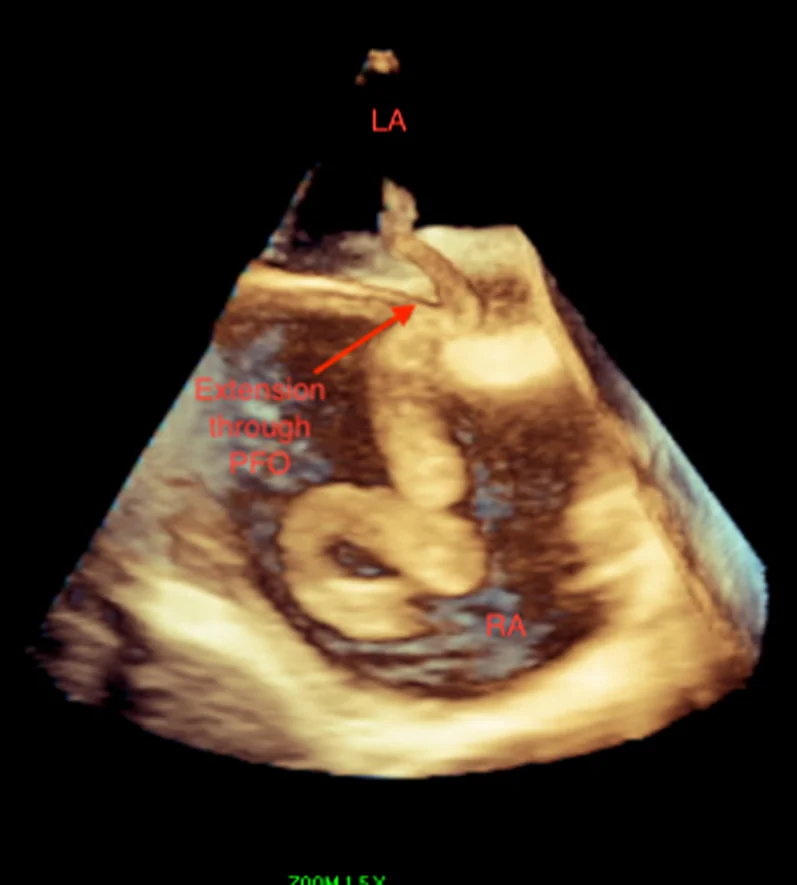
Three-Dimensional Transesophageal Echocardiogram View of Biatrial Thrombus Taken From Bicaval View, With Extension Through PFO Abbreviations as in Figure 1.

**Figure 3:**
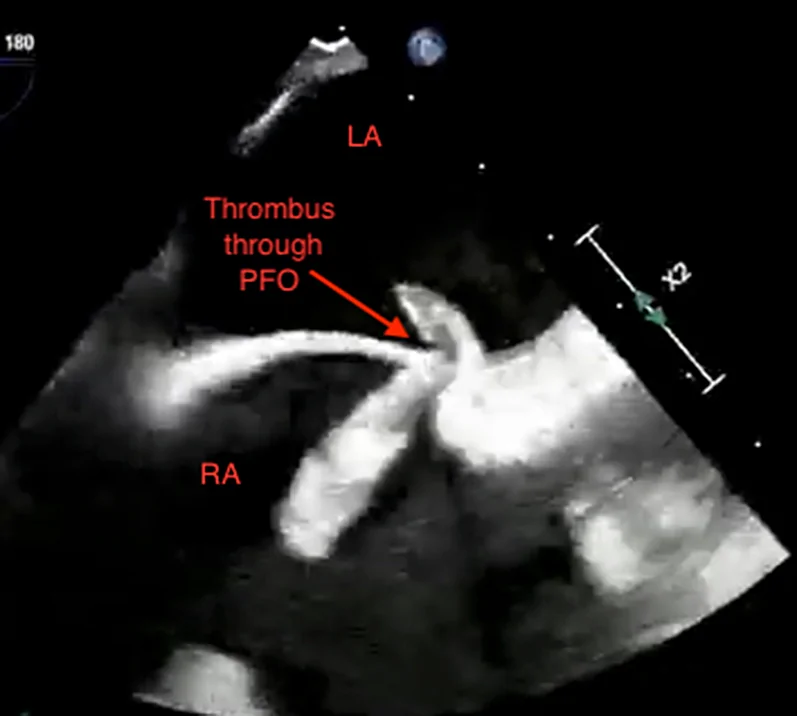
Biatrial Clot Extending Through a PFO Abbreviations as in Figure 1.

Computed tomographic angiography of the chest revealed an acute, large-volume saddle PE extending into the bilateral segmental and subsegmental pulmonary arteries with evidence of right heart strain (Figure 4). Bilateral lower extremity ultrasound showed a thrombus in the left proximal popliteal vein and in the gastrocnemius vein. Laboratory evaluation showed troponin <0.01 ng/mL. Liver enzymes were mildly elevated (AST 57 U/L, ALT 74 U/L, alkaline phosphatase 160 U/L), consistent with hepatic congestion.

**Figure 4:**
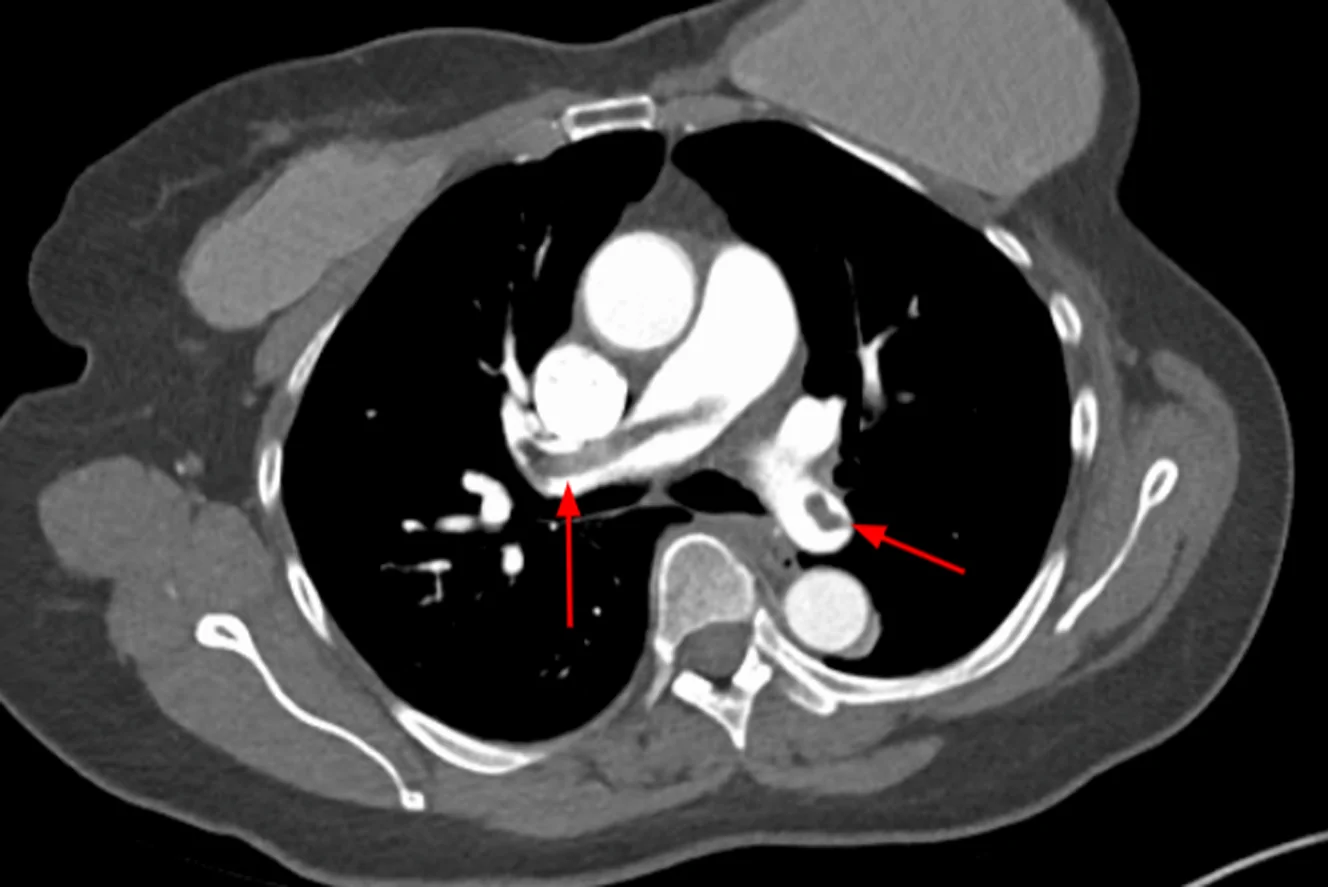
Computed Tomographic Angiography of the Chest Showing a Large Volume Acute Saddle Pulmonary Embolism

Screening mammogram just 3 months earlier was negative for any radiographic evidence of breast malignancy.

## Management

She was started on a heparin infusion and underwent emergent percutaneous mechanical thrombectomy under TEE guidance using a percutaneous aspiration thrombectomy system with interventional radiology (Video 4).

An 18-F perfusion cannula was placed in the left femoral vein. A 26-F Dry seal sheath was placed in the right internal jugular vein. Through this, the AngioVac (AngioDynamics) mechanical aspiration catheter was placed, and multiple passes at the intracardiac thrombus were made under TEE guidance with a cardiac anesthesiologist.

A 24-F sheath was then placed in the right femoral vein, and a pulmonary artery catheter was advanced through the RV and into the pulmonary artery. This catheter was exchanged for the Inari suction catheter, a large-bore suction embolectomy catheter system, separate from the aspiration catheter used for the transseptal thrombus. It was placed proximal to the clot, and utilizing self-expanding mesh and aspiration, extirpation was performed of the right and left pulmonary arteries.

A total of 14 cm of intracardiac thrombus was successfully removed (Figure 5), along with pulmonary emboli. She had an uncomplicated postprocedural course and was discharged after 5 days (including 1 day in the intensive care unit [ICU]) on oral anticoagulation.

**Figure 5:**
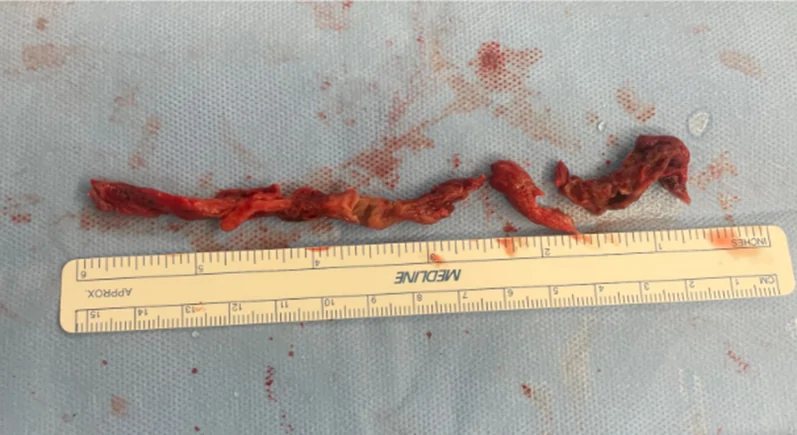
Extensive 14 cm Clot Removed From Atria and Ventricles Via Suction Thrombectomy With Good Patient Outcome

## Outcome and Follow-Up

Her neurological status and exam on discharge were the same as admission. Repeat transthoracic echocardiogram 3 weeks later demonstrated preserved ejection fraction (55%-60%), no residual atrial masses, and normalization of RV size and function. Hypercoagulable workup included prothrombin mutation, factor V Leiden mutation, antiphospholipid antibodies, lupus anticoagulant, antithrombin, and protein C and S deficiency testing, which was remarkable only for mild homocysteinemia (13.4 μmol/L), which has since normalized with vitamin supplementation. Since a screening mammogram was negative for breast cancer recurrence 3 months earlier and computed tomographic angiography imaging did not demonstrate any malignancy concerns, repeated testing was not pursued as malignancy recurrence was not thought to be contributing. Due to the unprovoked DVT/PE, she was advised to continue lifelong oral anticoagulation. She was referred to structural cardiology for possible intervention on the PFO; however, she was deemed not a candidate and, regardless, was not wanting to pursue further surgical intervention.

## Discussion

Clot in transit (CIT) represents an emergent manifestation of venous thromboembolism when thrombus enters the RA en route to the pulmonary arteries.^1^ In the presence of an atrial septal defect, or PFO, paradoxical embolization may occur. Clots in the RA traversing through an atrial septal defect/PFO into the LA are rare, but have been documented. Due to their infrequency, there is no consensus regarding optimal management. To our knowledge, successful extraction of a quadruple-chamber thrombus using percutaneous aspiration mechanical thrombectomy has not previously been described.

Surgical thrombectomy with PFO closure in low-risk surgical patients has been reported with variable outcomes.^2^^,^^3^ Reported complications include postoperative RV dysfunction requiring extracorporeal membrane oxygenation and progression from submassive to massive PE.^4^^,^^5^ Thrombolysis is generally reserved for hemodynamically unstable patients, but carries higher mortality risk and risk of embolization.^4^^,^^6^

Mechanical thrombectomy for PE without transseptal extension is increasingly used, particularly in elderly and higher-risk patients, because of lower mortality, fewer complications, and shorter hospital stays compared with surgery.^7^ Mechanical thrombectomy offers direct and immediate mechanical relief; however, it does not carry the need for thrombolytics.^8^ Surgical thrombectomy for PE without transseptal extension carries an average 22% mortality with a mean hospital stay of 10 days, 2 of those being in the ICU.^9^ In contrast, mechanical thrombectomy for PE without atrial clots has been associated with shorter hospitalizations and reduced ICU utilization.

Given the rarity and complexity of this presentation, consultation with cardiovascular surgery, cardiology, and interventional radiology was undertaken. She underwent emergent intracardiac mechanical thrombectomy via AngioVac percutaneous aspiration mechanical thrombectomy and PE thrombectomy via Inari suction catheter. After discussions with cardiovascular surgery, surgical thrombectomy was deferred because of distal, bilateral pulmonary emboli not amenable to surgical extraction. Additionally, it was thought surgical extraction of the pulmonary emboli requiring extracorporeal membrane oxygenation (ECMO) proposed a greater risk of RV failure given her already severe RV dysfunction seen on imaging in conjunction with her elevated pro-B-type natriuretic peptide. The extent of the clot and involvement of the left heart put the patient at risk for stroke, myocardial infarction, and other embolic phenomena as well. Mechanical thrombectomy would present these risks as well, but it was thought that the avoidance of circulatory arrest and bypass surgery made mechanical thrombectomy more favorable.

Given the uniqueness of a CIT of this type, and the novelty of the treatment, there is no direct study looking at the use of cerebral embolic protection devices in this scenario. Few case reports have been noted of the use in thrombectomy during the presence of PFO; however, no comparative or prospective studies exist. Further studies of thrombectomy in the presence of PFO while utilizing cerebral embolic protection devices are needed to determine whether their use significantly reduces the risks of clinical stroke.

Thrombectomy was favored over thrombolysis since she remained hemodynamically stable and without oxygen requirements, and thrombolysis alone poses an increased chance of embolic sequelae given the risk of thrombus fragmentation before complete thrombus elimination. As the fibrin structure of a clot weakens, thrombus pieces can fragment prior to the entire clot being eliminated.^4^ Since she was hemodynamically stable without oxygen requirements, thrombolytics were not indicated.

She has met with a structural cardiologist who was not recommending PFO closure given hematology’s life-long anticoagulation, theoretically reducing her risk of further clots combined with her homocysteinemia normalization. Additionally, her neurological function remained at her baseline throughout admission; therefore, it does not seem she suffered from any cerebral embolic phenomena that would have indicated PFO closure, and she was not wanting to pursue PFO intervention.

## Conclusions

In patients presenting with PE and CIT extending transeptally through a PFO, optimal management remains undefined. Surgical, mechanical, or thrombolytic approaches each carry significant risks.

This case demonstrates successful treatment of extensive biatrial and biventricular clot with saddle PE using peripheral aspiration mechanical thrombectomy, without clinically apparent systemic embolization or hemodynamic deterioration. As thrombectomy technology continues to evolve, further investigation is needed to better define indications and outcomes for mechanical vs surgical intervention in complex intracardiac thrombus.

**Visual Summary:**
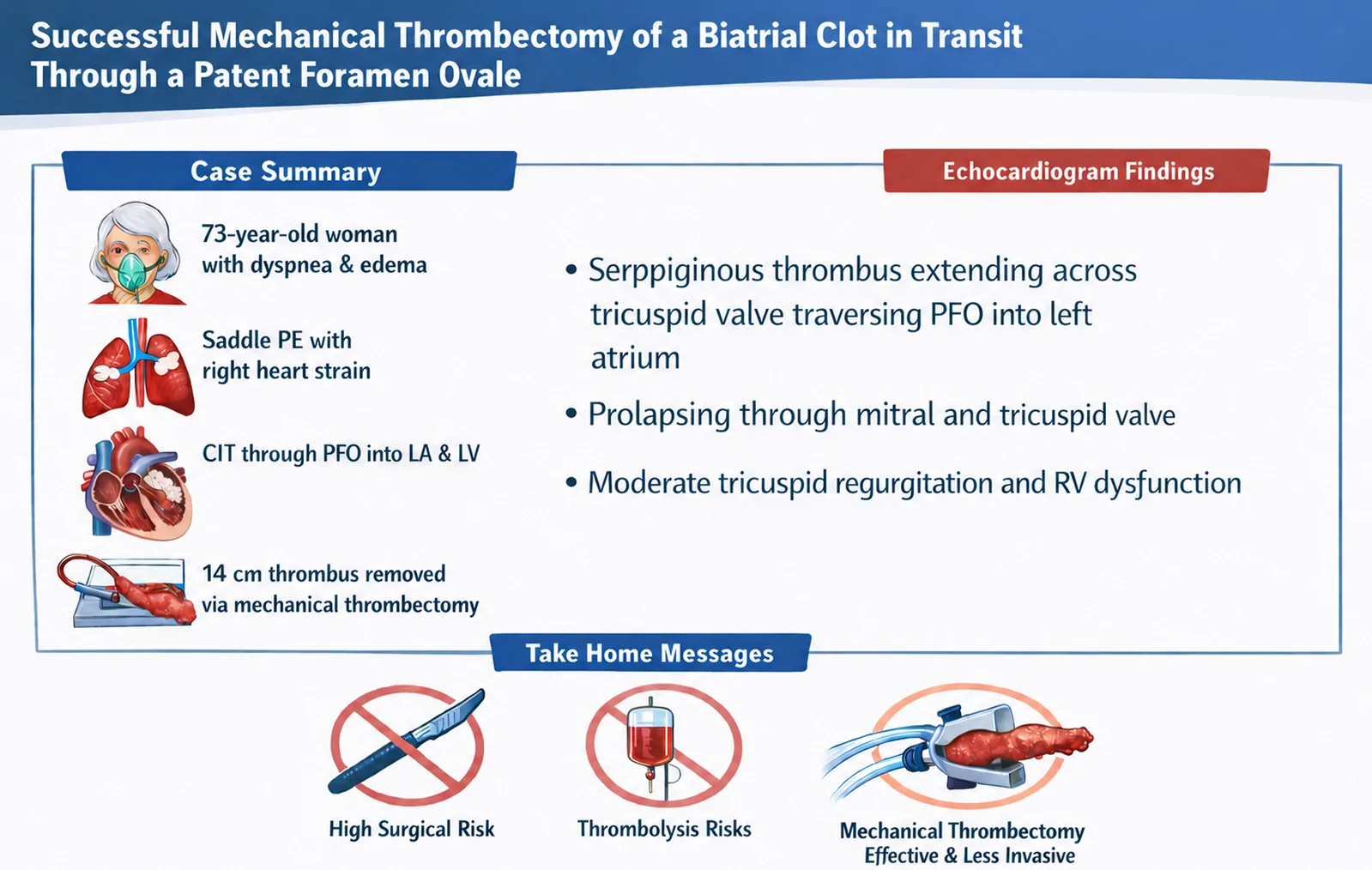
Case Summary and Takeaway Messages CIT = clot in transit; LA = left atrium; LV = left ventricle; PE = pulmonary embolism; PFO = patent foramen ovale; RV = right ventricle.

## Funding Support and Author Disclosures

The authors have reported that they have no relationships relevant to the contents of this paper to disclose.

**Take-Home Message**

• Mechanical thrombectomy may be an effective and less invasive option for complex biatrial/biventricular clot in transit with pulmonary embolism.

## Appendix

Video 1 Transthoracic Echocardiogram Apical 4-Chamber View With Biatrial Clot Prolapsing Across Mitral and Tricuspid Valves LA = left atrium; LV = left ventricle; PFO = patent foramen ovale; RA = right atrium; RV = right ventricle.

Video 2 Three-Dimensional Transthoracic Echocardiogram View of a Biatrial Thrombus in Right Atrium (RA), With Extension Through Patent Foramen Ovale (PFO)

Video 3 Mid-Esophageal Transthoracic Echocardiogram Showing Right Atrium (RA) Clot Prolapsing Through Tricuspid Valve Abbreviation as in Video 1.

Video 4 Bicaval Transthoracic Echocardiogram View Showing Engagement of the Atrial Clot With Suction Thrombectomy Catheter Abbreviation as in Video 1.
